# Serum Albumin is Linearly and Negatively Associated With the Risk of All-cause and Cardiovascular Death in Coronary Heart Disease Patients

**DOI:** 10.31083/RCM38034

**Published:** 2025-08-29

**Authors:** Jiayi Tong, Tao Wang, Qin Wei, Qing Hao, Fuchao Yu, Xuan Xu, Penghao Zhen

**Affiliations:** ^1^Department of Cardiology, Zhongda Hospital, School of Medicine, Southeast University, 210009 Nanjing, Jiangsu, China; ^2^Department of Cardiology, The First Affiliated Hospital of Soochow University, 215006 Suzhou, Jiangsu, China; ^3^State Key Laboratory of Bioelectronics, School of Biological Science and Medical Engineering, Southeast University, 211100 Nanjing, Jiangsu, China

**Keywords:** coronary heart disease, serum albumin, cardiovascular disease, cardiovascular death, all-cause death

## Abstract

**Background:**

Despite advances in treatment and the potential role of serum albumin as a prognostic biomarker, the mortality rate of individuals with coronary heart disease (CHD) continues to increase. Thus, this study aimed to assess the relationship between serum albumin levels and the risk of all-cause mortality and cardiovascular death in individuals with CHD.

**Methods:**

This large-scale retrospective cohort study included 1556 participants diagnosed with CHD from the National Health and Nutrition Examination Survey spanning 1999 to 2015. We conducted multivariate Cox regression, subgroup and sensitivity analyses, and restricted cubic spline (RCS) plots to examine the link between serum albumin levels and all-cause mortality and cardiovascular death.

**Results:**

After gradually adjusting the confounding variables, serum albumin consistently demonstrated a strong link to increased overall and cardiovascular-related mortality risk when employed as a continuous variable (hazard ratio [HR]: 0.938, 95% confidence interval [CI]: 0.912–0.964; *p* < 0.001; HR: 0.921, 95% CI: 0.884–0.960; *p* < 0.001; respectively); meanwhile, serum albumin as a three-category variable, with Tertile 1 (T1, ≤40 g/L), Tertile 2 (T2, 40–43 g/L), and Tertile 3 (T3, >43 g/L), was only closely related to the risk of all-cause death (T2 vs. T1, HR: 0.771, 95% CI: 0.633–0.939; *p* = 0.010; T3 vs. T1, HR: 0.761, 95% CI: 0.612–0.947; *p* = 0.014; respectively). Subgroup analysis showed that serum albumin was linked to all-cause mortality across most groups (≤60 or >60 years, male or female, and without hypertension, diabetes, or chronic kidney disease); however, its correlation with cardiovascular death was observed only in the subgroup without hypertension (*p* < 0.05). The sensitivity analysis indicated that excluding participants with an estimated glomerular filtration rate <30 mL/min/1.73 m^2^ did not alter the association between serum albumin and the risk of all-cause and cardiovascular mortality. Moreover, the RCS analysis further supported a consistent negative linear trend between serum albumin levels and mortality risks (*p* for nonlinearity >0.05).

**Conclusions:**

The serum albumin levels in individuals with CHD were inversely and linearly related to all-cause mortality and cardiovascular death risk.

## 1. Introduction

Cardiovascular disease (CVD) has become one of the most important public health problems in the world, and it is also one of the most important causes of death in the world, among which the prevalence and mortality of coronary heart disease (CHD) are the highest [1, 2]. Although the medical treatment and surgical treatment of CHD have become standardized, the prognosis is still not optimistic, and the mortality rate is still gradually rising in patients with CHD [3]. Therefore, screening and early intervention of reversible risk factors is very important to improve the prognosis and quality of life of patients with CHD.

Serum albumin is the most abundant multifunctional protein in the blood. It not only plays a vital role in the regulation of colloid osmotic pressure, but also has antioxidant properties, and can also respond to various diseases as an acute phase reaction protein [4, 5, 6, 7]. However, unlike other acute phase reactive proteins, its concentration is at a low level in the acute phase [5]. In addition, albumin is a biomarker reflecting nutritional status. Hypoalbuminemia is due mainly to the decline in liver synthesis, decreased intake and chronic inflammation, which can be seen in many diseases [8, 9]. Recent research has indicated that serum albumin is strongly associated with the outcome and mortality of many chronic diseases, including cirrhosis, chronic heart failure and chronic obstructive pulmonary disease [10, 11, 12, 13, 14, 15]. Moreover, serum albumin and its composite indicators play a significant role in assessing cardiovascular risk and predicting clinical outcomes [16]. Several studies have demonstrated that serum albumin, whether used alone or in combination with other clinical parameters, is strongly associated with no-reflow and new-onset atrial fibrillation following percutaneous coronary intervention in patients with acute myocardial infarction [17, 18]. It has also been linked to left ventricular hypertrophy in individuals with hypertension [19]. Additionally, lower albumin levels have been shown to predict long-term all-cause mortality in patients with dual-chamber permanent pacemakers [20]. However, the relationship between serum albumin and mortality risk in patients with coronary heart disease remains unclear.

Therefore, based on the current research background and knowledge gaps, and to provide therapeutic strategies for the management of albumin in the prognosis of CHD, we used data from the 1999–2015 National Health and Nutrition Examination Survey (NHANES), to examine the association between serum albumin levels and the death risks for CHD patients.

## 2. Methods

### 2.1 Research Design and Participants

**Fig. 1.**
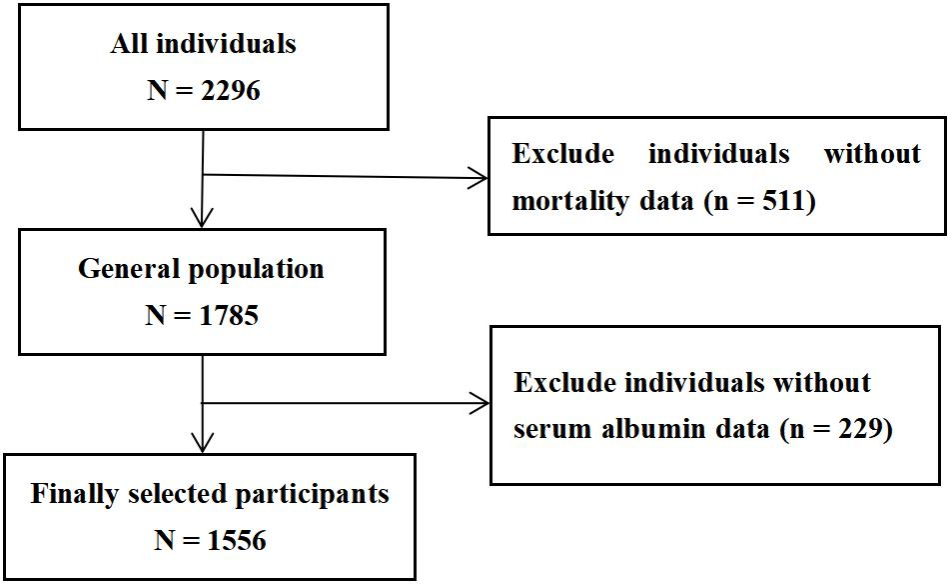
**Flow chart of selected participants**.

As outlined in Fig. 1, based on data from the NHANES between 1999 and 2015, a total of 1556 individuals were included. Inclusion criteria: (1) Adults aged ≥18 years; (2) Self-reported diagnosis of CHD in the NHANES database. Exclusion criteria: (1) Individuals without information on mortality status or time; (2) Individuals lacking baseline laboratory data on serum albumin. The protocol was approved by the Institutional Review Board of the National Center for Health Statistics (Protocol #98-12, #2005-06) and complied with the Declaration of Helsinki. All participants provided informed consent before participating in the study.

### 2.2 Data Source and Initial Classification

This research is a retrospective cohort study based on cross-sectional NHANES data linked to mortality records from the National Death Index (NDI). The fundamental step was gathering baseline data, which was carried out in line with the pre-determined research design. The collected data covered various aspects, including demographic information, physical examination data, details about complications, drug treatment records, and biomarker data. For instance, smoking status was simply categorized as a binary variable—whether an individual was currently smoking or not.

### 2.3 Medical Condition Definitions

Hypertension was defined by a documented diagnosis, elevated systolic blood pressure (SBP ≥140 mmHg) and/or diastolic blood pressure (DBP ≥90 mmHg), or the administration of antihypertensive therapy. A diagnosis of diabetes was based on physician confirmation, elevated fasting plasma glucose (FPG ≥7.0 mmol/L), increased hemoglobin A1c (HbA1c ≥6.5%), or ongoing antidiabetic treatment. Hypercholesterolemia was defined based on a previous diagnosis or ongoing use of cholesterol-lowering drugs. A self-reported history of stroke was used to define stroke. The estimated glomerular filtration rate (eGFR) was calculated using established methods from the literature, and chronic kidney disease (CKD) was defined as eGFR less than 60 mL/min/1.73 m^2^ [21, 22].

### 2.4 Follow-up and Outcome Measurement

Participants in the study were tracked from the date of their initial NHANES interview until December 31, 2015. During this follow-up period, the outcomes assessed were all-cause mortality and cardiovascular death, which were classified using ICD-10 codes. This period allowed for the collection of comprehensive data on mortality risks, offering valuable insights into the long-term health implications of the variables under study. The use of ICD-10 coding ensured standardized and reliable classification of the causes of death, which was essential for the analysis of mortality patterns in the cohort.

### 2.5 Approach to Statistical Evaluation

For this analysis, participants were grouped based on serum albumin tertiles: T1 (≤40 g/L), T2 (40–43 g/L), and T3 (>43 g/L). Before comparing continuous variables across these groups, we assessed the normality of their distributions using the Shapiro-Wilk test. Variables that followed a normal distribution were compared using one-way ANOVA, while non-normally distributed variables were analyzed using Kruskal-Wallis H tests. Categorical variables were analyzed using the Chi-square test to assess differences between groups. Survival probabilities for all-cause and cardiovascular mortality were estimated using Kaplan-Meier survival curves across the three serum albumin groups. To explore the association between serum albumin and outcomes, Cox regression models were utilized. Model 1 was unadjusted; Model 2 was adjusted for age and sex; Model 3 was further adjusted for a comprehensive set of covariates depending on the outcome. For all-cause mortality, Model 3 included age, sex, diabetes, hypercholesterolemia, stroke, hypoglycemic drugs, cholesterol-lowering drugs, body mass index (BMI), SBP, DBP, white blood cell count, hemoglobin, eGFR, fibrinogen, C-reactive protein (CRP), FPG, and HbA1c. For cardiovascular mortality, Model 3 included age, sex, hypertension, diabetes, stroke, hypoglycemic drugs, SBP, DBP, white blood cell count, hemoglobin, platelets, alanine aminotransferase (ALT), eGFR, fibrinogen, and CRP. Subgroup and sensitivity analyses were conducted to evaluate the stability and reliability of the relationship between serum albumin and the mortality risks. Specifically, sensitivity analysis was performed by excluding participants with an eGFR <30 mL/min/1.73 m^2^ to assess whether severe renal impairment affected the observed associations. Additionally, restricted cubic spline (RCS) plots with three knots were employed to examine potential nonlinear associations between serum albumin levels and both all-cause and cardiovascular death, and these models were fully adjusted for covariates (i.e., based on Model 3). Missing data were handled using a combination of multiple imputation for variables with low rates of missingness and predefined exclusion criteria for individuals with missing key variables, such as serum albumin or mortality data, to minimize bias and maintain data integrity. All statistical analyses were performed using SPSS version 26.0 (IBM Corp., Armonk, NY, USA) and R version 4.1.3 (R Foundation for Statistical Computing, Vienna, Austria), with statistical significance defined by a *p*-value of less than 0.05.

## 3. Results

### 3.1 Participant Characteristics at Baseline

**Fig. 2.**
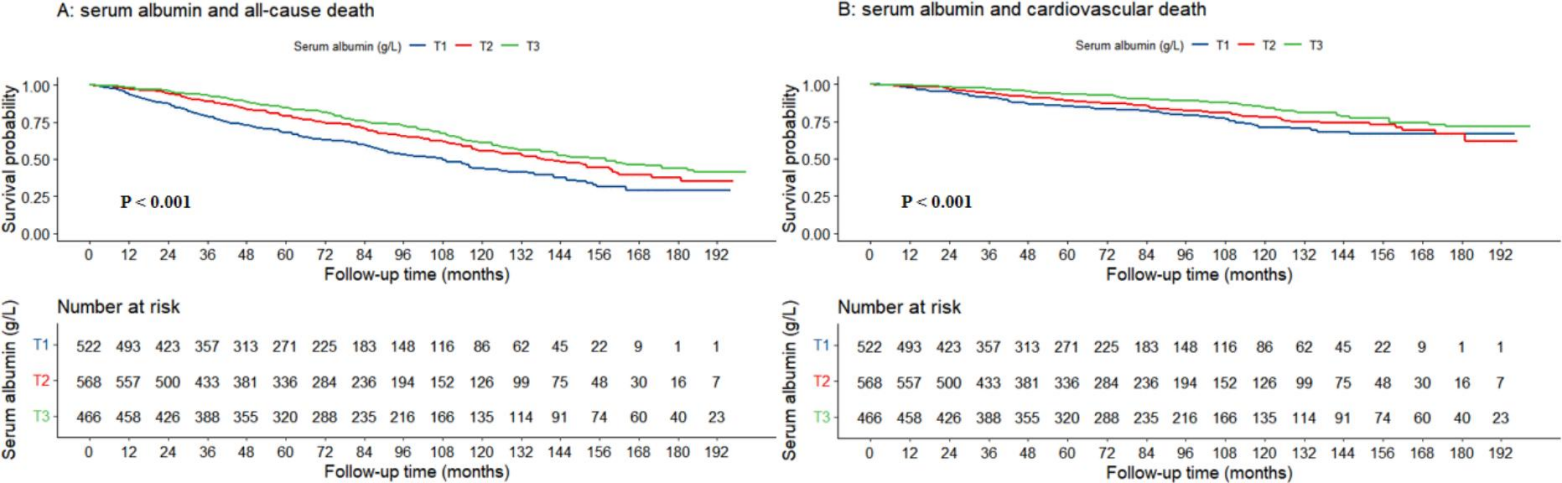
**The association between serum albumin levels and all-cause mortality (A) as well as cardiovascular death (B) shown through Kaplan-Meier survival curves**.

As presented in Table 1, individuals with higher serum albumin levels were younger, were more likely to be males, had a lower prevalence of diabetes and all-cause mortality, uses significantly less hypoglycemic drugs, had lower BMI, lower levels of white blood cell, platelets, fibrinogen, CRP, FPG, HbA1c, and higher levels of DBP, triglycerides, total cholesterol, hemoglobin, abnormal liver function and eGFR (*p* < 0.05). The probability for all-cause and cardiovascular mortality decreased over time in all three serum albumin groups (Fig. 2). At each follow-up period, the T3 group demonstrated higher survival probabilities than the T1 group (*p* < 0.001).

**Table 1.** **Baseline characteristics**.

		All participants	T1 (≤40 g/L)	T2 (40–43 g/L)	T3 (>43 g/L)	*p* value
N		1556	522	568	466	
Age, years		68.66 ± 11.56	69.58 ± 11.04	69.20 ± 11.01	66.96 ± 12.59	0.001
Sex, male, n (%)		1048 (67.40)	316 (60.50)	388 (68.30)	344 (73.80)	<0.001
Smoking, n (%)						0.110
	Yes	268 (17.20)	109 (20.90)	89 (15.70)	70 (15.00)	
	No	735 (47.20)	236 (45.20)	271 (47.70)	228 (48.90)	
	Missing	553 (35.50)	177 (33.90)	208 (36.60)	168 (36.10)	
Comorbidities, n (%)						
Hypertension						0.094
	Yes	1125 (72.30)	394 (75.50)	405 (71.30)	326 (70.00)	
	No	425 (27.30)	124 (23.80)	162 (28.50)	139 (29.80)	
	Missing	6 (0.40)	4 (0.80)	1 (0.20)	1 (0.20)	
Diabetes						<0.001
	Yes	491 (31.60)	204 (39.10)	178 (31.30)	109 (23.40)	
	No	1063 (68.30)	318 (60.90)	390 (68.70)	355 (76.20)	
	Missing	2 (0.10)	0 (0)	0 (0)	2 (0.40)	
Hypercholesterolemia						0.315
	Yes	1046 (67.20)	336 (64.40)	395 (69.50)	315 (67.60)	
	No	429 (27.60)	152 (29.10)	147 (25.90)	130 (27.90)	
	Missing	81 (5.20)	34 (6.50)	26 (4.60)	21 (4.50)	
Stroke						0.162
	Yes	238 (15.30)	92 (17.60)	89 (15.70)	57 (12.20)	
	No	1316 (84.60)	429 (82.20)	478 (84.20)	409 (87.80)	
	Missing	2 (0.10)	1 (0.20)	1 (0.10)	0 (0)	
Treatment, n (%)						
Hypotensive drugs						0.266
	Yes	1025 (65.90)	358 (68.60)	367 (64.60)	300 (64.40)	
	No	476 (30.60)	144 (27.60)	178 (31.30)	154 (33.00)	
	Missing	55 (3.50)	20 (3.80)	23 (4.00)	12 (2.60)	
Hypoglycemic drugs						<0.001
	Yes	458 (29.40)	197 (37.70)	165 (29.00)	96 (20.60)	
	No	1097 (70.50)	325 (62.30)	403 (71.00)	369 (79.20)	
	Missing	1 (0.10)	0 (0)	0 (0)	1 (0.20)	
Cholesterol-lowering drugs						0.167
	Yes	916 (58.90)	291 (55.70)	357 (62.90)	268 (57.50)	
	No	478 (30.70)	170 (32.60)	158 (27.80)	150 (32.20)	
	Missing	162 (10.40)	61 (11.70)	53 (9.30)	48 (10.30)	
Body mass index, kg/m^2^		29.41 ± 6.14	30.59 ± 7.24	29.40 ± 5.78	28.13 ± 4.87	<0.001
Systolic blood pressure, mmHg		132.69 ± 21.87	132.32 ± 22.17	132.06 ± 21.79	133.85 ± 21.64	0.404
Diastolic blood pressure, mmHg		66.97 ± 12.62	65.00 ± 12.71	67.17 ± 12.27	68.83 ± 12.65	<0.001
Triglycerides, mmol/L		1.46 (1.03, 2.15)	1.40 (0.95, 2.06)	1.60 (1.14, 2.32)	1.67 (1.16, 2.33)	0.020
Total cholesterol, mmol/L		4.69 ± 1.22	4.55 ± 1.20	4.71 ± 1.23	4.82 ± 1.21	0.002
LDL‑C, mmol/L		2.58 ± 0.96	2.49 ± 0.95	2.60 ± 0.92	2.66 ± 1.01	0.150
HDL‑C, mmol/L		1.24 ± 0.37	1.24 ± 0.36	1.23 ± 0.38	1.25 ± 0.37	0.777
White blood cell, ×10^9^/L		7.42 ± 2.57	7.63 ± 2.28	7.44 ± 3.13	7.18 ± 2.04	0.023
Hemoglobin, g/dL		14.03 ± 1.60	13.43 ± 1.68	14.13 ± 1.46	14.58 ± 1.43	<0.001
Platelets, ×10^9^/L		227.91 ± 68.96	234.26 ± 74.23	227.28 ± 66.74	221.56 ± 64.89	0.015
Alanine transaminase, U/L		21.00 (16.00, 27.00)	19.00 (15.00, 25.00)	21.50 (18.00, 26.25)	24.00 (18.00, 30.00)	<0.001
Aspartate aminotransferase, U/L		24.00 (20.00, 28.00)	22.00 (19.00, 27.00)	23.50 (20.00, 28.25)	26.00 (22.00, 31.00)	<0.001
Total bilirubin, umol/L		12.74 ± 5.40	11.79 ± 4.90	12.87 ± 5.45	13.63 ± 5.70	<0.001
eGFR, mL/min/1.73 m^2^		77.79 ± 29.64	70.80 ± 29.60	78.94 ± 27.85	84.23 ± 30.20	<0.001
Fibrinogen, g/L		4.03 ± 0.85	4.48 ± 0.94	3.99 ± 0.81	3.80 ± 0.73	<0.001
C-reactive protein, mg/L		0.26 (0.12, 0.61)	0.39 (0.15, 0.92)	0.25 (0.11, 0.52)	0.20 (0.08, 0.37)	<0.001
Fasting plasma glucose, mmol/L		6.85 ± 2.62	7.13 ± 3.30	6.95 ± 2.53	6.37 ± 1.55	0.005
Hemoglobin A1c, %		6.20 ± 1.26	6.48 ± 1.52	6.17 ± 1.16	5.92 ± 0.94	<0.001
Outcomes, n (%)						
All-cause death						0.001
	Yes	604 (38.80)	238 (45.60)	201 (35.40)	165 (35.40)	
	No	952 (61.20)	284 (54.40)	367 (64.60)	301 (64.60)	
Cardiovascular death						0.174
	Yes	244 (15.70)	92 (17.60)	90 (15.80)	62 (13.30)	
	No	1312 (84.30)	430 (82.40)	478 (84.20)	404 (86.70)	

Missing values for smoking, comorbidities, and medication use were shown in the table. LDL-C, low-density lipoprotein cholesterol; HDL-C, high-density lipoprotein cholesterol; eGFR, estimated glomerular filtration rate.

### 3.2 Multivariate Adjusted Association

As shown in Table 2. In the unadjusted model 1, the Cox regression analysis indicated that elevated serum albumin levels were significantly associated with a reduced risk of both all-cause and cardiovascular death. This relationship was observed whether albumin was analyzed as a continuous or categorized variable. For continuous measurements, the hazard ratios (HR) were 0.909 (95% CI: 0.889–0.930, *p* < 0.001) and 0.904 (95% CI: 0.873–0.936, *p* < 0.001). When classified into tertiles, the T3 group (highest albumin) had a lower hazard ratio compared to the T1 group (lowest albumin), with HRs of 0.543 (95% CI: 0.444–0.663, *p* < 0.001) and 0.532 (95% CI: 0.385–0.737, *p* < 0.001), respectively. After adjusting for age and sex (model 2), serum albumin remained significantly related to reduced risks of all-cause and cardiovascular death. The HRs were 0.908 (95% CI: 0.886–0.930, *p* < 0.001) and 0.905 (95% CI: 0.871–0.940, *p* < 0.001) when as a continuous variable. When treated as a categorical variable, the T3 group compared to T1 had HRs of 0.589 (95% CI: 0.482–0.720, *p* < 0.001) and 0.580 (95% CI: 0.418–0.803, *p* = 0.001), respectively. After accounting for all confounding factors in Model 3, serum albumin as a continuous variable remained significantly associated with all-cause and cardiovascular death (HR: 0.938, 95% CI: 0.912–0.964, *p* < 0.001; HR: 0.921, 95% CI: 0.884–0.960, *p* < 0.001). However, when classified into three categories, serum albumin was only significantly linked to all-cause death. Comparisons of the various groups showed that the T2 group relative to T1 had an HR of 0.771 (95% CI: 0.633–0.939, *p* = 0.010), and the T3 group compared to T1 had an HR of 0.761 (95% CI: 0.612–0.947, *p* = 0.014).

**Table 2.** **Cox regression analysis of serum albumin with all-cause and cardiovascular death**.

		Model 1		Model 2		Model 3	
		HR (95% CI)	*p* value	HR (95% CI)	*p* value	HR (95% CI)	*p* value
All-cause death							
	T1	Ref.	-	Ref.	-	Ref.	-
	T2	0.659 (0.546, 0.795)	<0.001	0.676 (0.560, 0.816)	<0.001	0.771 (0.633, 0.939)	0.010
	T3	0.543 (0.444, 0.663)	<0.001	0.589 (0.482, 0.720)	<0.001	0.761 (0.612, 0.947)	0.014
	per 1 unit increment	0.909 (0.889, 0.930)	<0.001	0.908 (0.886, 0.930)	<0.001	0.938 (0.912, 0.964)	<0.001
Cardiovascular death							
	T1	Ref.	-	Ref.	-	Ref.	-
	T2	0.765 (0.571, 1.023)	0.071	0.785 (0.587, 1.050)	0.103	0.893 (0.658, 1.211)	0.466
	T3	0.532 (0.385, 0.737)	<0.001	0.580 (0.418, 0.803)	0.001	0.734 (0.518, 1.040)	0.082
	per 1 unit increment	0.904 (0.873, 0.936)	<0.001	0.905 (0.871, 0.940)	<0.001	0.921 (0.884, 0.960)	<0.001

HR, hazard ratio.

### 3.3 Subgroup Analysis, Sensitivity Analysis and RCS Analysis

**Fig. 3.**
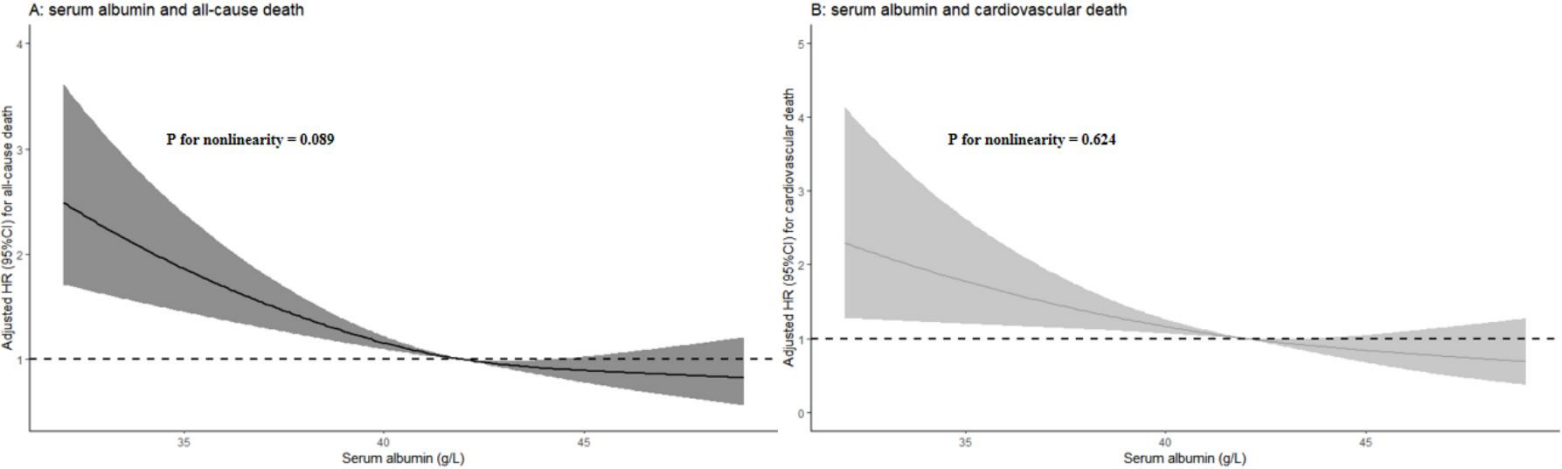
**Restricted cubic spline plots showing the relationship between serum albumin levels and all-cause (A) as well as cardiovascular death (B)**.

Subgroup analysis of Table 3 revealed that serum albumin was linked to the likelihood of mortality from all causes across several subgroups, including participants aged ≤60 or >60 years, males, and those without hypertension, diabetes, or CKD. Notably, in the female subgroup, the significant association with all-cause mortality was observed in the T2 group rather than the T3 group. However, the relationship between serum albumin and cardiovascular death was only observed in individuals without hypertension (T2 vs T1, HR: 0.480, 95% CI: 0.249–0.923, *p* < 0.05), and not in T3, which should be noted to avoid misinterpretation. In sensitivity analyses, the exclusion of participants with eGFR <30 mL/min/1.73 m^2^ did not alter the previously observed correlation. The relationship between serum albumin and both all-cause and cardiovascular mortality remained in line with the observed results from the three models shown in Table 2 (Table 4). Further analysis using RCS showed that serum albumin levels were linearly and inversely related to all-cause and cardiovascular death (nonlinearity *p*-values = 0.089 and 0.624, respectively) (Fig. 3).

**Table 3.** **Subgroups analysis**.

		All-cause death						Cardiovascular death					
		T1	T2		T3			T1	T2		T3		
		Ref.	HR (95% CI)	*p* value	HR (95% CI)	*p* value	*p* trend	Ref.	HR (95% CI)	*p* value	HR (95% CI)	*p* value	*p* trend
Age													
	≤60 years	1.0	0.375 (0.184, 0.765)	0.007	0.442 (0.228, 0.859)	0.016	0.012	1.0	0.879 (0.266, 2.909)	0.833	1.099 (0.300, 4.019)	0.887	0.936
	>60 years	1.0	0.816 (0.663, 1.005)	0.055	0.766 (0.607, 0.966)	0.025	0.052	1.0	0.913 (0.664, 1.255)	0.575	0.721 (0.500, 1.040)	0.080	0.203
Sex													
	Male	1.0	0.815 (0.641, 1.035)	0.094	0.717 (0.548, 0.937)	0.015	0.045	1.0	1.045 (0.717, 1.523)	0.819	0.844 (0.555, 1.284)	0.429	0.536
	Female	1.0	0.641 (0.440, 0.934)	0.021	0.899 (0.602, 1.343)	0.603	0.060	1.0	0.659 (0.370, 1.174)	0.157	0.563 (0.281, 1.130)	0.106	0.185
Hypertension													
	Yes	1.0	0.789 (0.621, 1.003)	0.053	0.772 (0.593, 1.005)	0.055	0.081	1.0	0.975 (0.684, 1.390)	0.890	0.837 (0.562, 1.248)	0.383	0.647
	No	1.0	0.539 (0.374, 0.777)	0.001	0.572 (0.387, 0.847)	0.005	0.002	1.0	0.480 (0.249, 0.923)	0.028	0.480 (0.230, 1.003)	0.051	0.054
Diabetes													
	Yes	1.0	0.787 (0.563, 1.099)	0.159	0.732 (0.490, 1.092)	0.126	0.224	1.0	0.944 (0.572, 1.560)	0.824	0.650 (0.338, 1.247)	0.195	0.405
	No	1.0	0.749 (0.582, 0.965)	0.026	0.744 (0.569, 0.973)	0.031	0.041	1.0	0.942 (0.638, 1.390)	0.762	0.844 (0.555, 1.284)	0.427	0.723
Chronic kidney disease													
	Yes	1.0	0.734 (0.527, 1.022)	0.067	0.790 (0.545, 1.145)	0.214	0.150	1.0	0.759 (0.456, 1.264)	0.289	0.855 (0.485, 1.505)	0.587	0.560
	No	1.0	0.759 (0.587, 0.982)	0.036	0.706 (0.532, 0.938)	0.016	0.037	1.0	0.997 (0.666, 1.494)	0.987	0.717 (0.451, 1.140)	0.159	0.225

**Table 4.** **Sensitivity analysis after excluding individuals with eGFR <30 mL/min/1.73 m^2^**.

		Model 1		Model 2		Model 3	
		HR (95% CI)	*p* value	HR (95% CI)	*p* value	HR (95% CI)	*p* value
All-cause death							
	T1	Ref.	-	Ref.	-	Ref.	-
	T2	0.678 (0.557, 0.825)	<0.001	0.691 (0.567, 0.840)	<0.001	0.740 (0.603, 0.908)	0.004
	T3	0.562 (0.456, 0.692)	<0.001	0.602 (0.488, 0.742)	<0.001	0.726 (0.580, 0.908)	0.005
	*p* for trend	-	<0.001	-	<0.001	-	0.005
	per 1 unit increment	0.914 (0.892, 0.935)	<0.001	0.914 (0.891, 0.937)	<0.001	0.932 (0.906, 0.959)	<0.001
Cardiovascular death							
	T1	Ref.	-	Ref.	-	Ref.	-
	T2	0.798 (0.588, 1.083)	0.147	0.828 (0.610, 1.123)	0.225	0.911 (0.662, 1.255)	0.570
	T3	0.555 (0.396, 0.779)	0.001	0.612 (0.436, 0.859)	0.005	0.734 (0.511, 1.055)	0.095
	*p* for trend	-	0.002	-	0.016	-	0.236
	per 1 unit increment	0.913 (0.880, 0.948)	<0.001	0.912 (0.876, 0.950)	<0.001	0.923 (0.885, 0.962)	<0.001

## 4. Discussion

In this large, retrospective cohort study, our findings indicated that serum albumin was significantly associated with both all-cause mortality and cardiovascular death among those suffering from CHD. Moreover, this association presented a linear negative correlation pattern. These results not only emphasized the significance of serum albumin in the prognostic assessment and care of CHD patients, but also suggested that for individuals with hypoalbuminemia, appropriate supplementation of exogenous albumin might have potential clinical value in improving the prognosis of patients with CHD and even CVD. However, whether higher concentrations of serum albumin will improve patient outcomes will require further study to identify those subgroups of patients who will benefit most from this therapy.

Several studies have verified the relationship between serum albumin and the prognosis of certain chronic diseases. In a clinical trial involving 559 participants, Feng *et al*. [10] demonstrated an independent negative correlation between 4-year all-cause mortality and baseline serum albumin levels in heart failure patients. In a cohort study of 3398 patients with severe chronic obstructive pulmonary disease, Ling *et al*. [11] identified a negative relationship between serum albumin levels and in-hospital mortality. In a case-control study of 1383 patients with nonalcoholic fatty liver diagnosed hepatic biopsies, Takahashi *et al*. [23] found that individuals with moderate or lower serum albumin levels had a higher likelihood of death or requiring a liver transplant compared to those with higher serum albumin levels. Another large prospective cohort study also reported that acute inpatients with serum albumin levels ≤3.4 g/dL faced a greater risk of all-cause mortality or ischemic events compared to those with serum albumin levels >3.4 g/dL [24]. In addition, two recent prospective single-center studies in the emergency department of an Italian hospital showed that the serum albumin level measured on admission could independently predict the mortality of patients with acute infection 30 days after discharge, and that serum albumin had a higher predictive value for 30-day mortality in patients with a low to medium organ failure assessment score [25, 26]. Thomas *et al*. [27] found that reduced albumin levels were independently linked to higher mortality rates 5 and 9 years after follow-up in 331 patients over 55 of age with an intra-capsular fracture of the femoral neck, and they identified 42 g/L as the optimal threshold of serum albumin for predicting patient survival, these results were helpful for decision-making in patients undergoing total hip replacement or hemiarthroplasty. In addition, in a prospective cohort study involving 1000 patients with ischemic stroke, higher serum albumin levels were significantly linked to a lower risk of death, emphasizing its potential clinical role as a prognostic marker in this patient group [28]. While these studies did not include CHD patients, our findings align with their results, confirming a strong association between serum albumin levels and the risk of both all-cause and cardiovascular mortality. These studies added further evidence for the detrimental effects of hypoalbuminemia in CVD, and that low serum albumin should be considered as a risk factor for increased mortality in patients with CHD. However, it is still unknown what the safe concentration of serum albumin should be in patients with CHD. A recent study questioned the safety of higher the serum albumin levels. In the PRACTICE study, a 60-month follow-up of 14,994 coronary artery disease patients revealed a U-shaped relationship between serum albumin and the risks of major adverse cardiovascular events. After adjusting for confounders, albumin levels below 45 g/L were linked to lower risks, while levels above 50 g/L were associated with higher risks [29]. They found that albumin levels below 35 g/L were independently related to higher all-cause and cardiac death risk [29]. In addition to its role as an individual biomarker, serum albumin also contributes to several composite indices that have shown strong associations with poor outcomes in cardiopulmonary diseases. Among these, the prognostic nutritional index (PNI) and the Naples scoring systems have drawn increasing attention. Forexample, in a study involving 221 patients with heart failure who had implantable cardioverter-defibrillators, reported that lower PNI scores were significantly linked to higher all-cause mortality [30]. Likewise, Şaylık *et al*. [31], in a cohort of 1889 patients with acute ST-segment elevation myocardial infarction, found that the Naples Score independently predicted long-term mortality and improved the prognostic accuracy beyond traditional models. Another study also demonstrated that the Naples prognostic score was an independent predictor of long-term mortality in patients with acute pulmonary embolism [32]. These findings collectively suggest that serum albumin, whether assessed on its own or as part of a composite risk score, holds substantial prognostic value in the context of CVD. It is worth noting that while a significant association was observed, causality cannot be inferred from this observational study. Serum albumin may not be a direct causal factor but rather a surrogate marker of underlying pathophysiological conditions, such as chronic inflammation, malnutrition, or systemic illness. Hypoalbuminemia could reflect a catabolic state, liver dysfunction, or ongoing inflammatory processes, all of which may contribute to poor outcomes in CHD patients. This highlights the importance of interpreting our findings within the broader clinical and biological context. Besides, subgroup analysis revealed a more pronounced inverse association between serum albumin levels and both all-cause and cardiovascular mortality in non-hypertensive individuals compared to those with hypertension, suggesting a potentially meaningful but borderline significant protective effect. This pattern may reflect the complex interplay between hypertension, vascular damage, albuminuria, and systemic inflammation, all of which could modulate the prognostic value of serum albumin. Further investigation is warranted to understand how hypertension-related pathophysiology might attenuate the protective association of albumin in CHD patients.

Our study has several limitations. First, the study population reflected a small percentage of the American population. Second, due to the limitations of population survey data, the research data did not include echocardiography and coronary angiography data. Thirdly, due to the limitation of the database, we failed to investigate the association between serum albumin and additional outcomes of individuals with CHD, such as major adverse cardiovascular events. Fourth, serum albumin levels were measured at baseline during the NHANES survey, while CHD diagnosis was based on self-reported or physician-confirmed history, meaning that albumin measurements may have occurred after the onset of CHD. This raises the possibility of reverse causality—where CHD itself may contribute to lower albumin levels through mechanisms such as chronic inflammation or malnutrition—potentially biasing the observed associations. Fifth, due to the limitations of the NHANES dataset, we were unable to compare serum albumin with other established cardiovascular biomarkers, such as B-type natriuretic peptide or high-sensitivity troponin, which were not systematically collected. This limits our ability to evaluate the relative prognostic value of albumin in the context of risk stratification. We have acknowledged this as a limitation and suggest that future studies incorporate a wider range of biomarkers for a more comprehensive assessment. Sixth, although our study covered a 16-year period, the NHANES dataset does not provide dynamic measures of serum albumin or detailed records of evolving CHD management strategies over time. As a result, we were unable to assess whether secular trends in albumin levels or treatment practices influenced the observed associations. We have acknowledged this limitation and suggest that future research based on long-term follow-up data is needed to explore the potential impact of temporal changes. Finally, we might have overlooked some potential risk factors, such as genetic susceptibility, socioeconomic status, dietary nutrition, and environmental factors, associated with increased risk in CHD patients.

## 5. Conclusions

In this retrospective cohort study utilizing the survey data from the general population, we discovered not only that serum albumin levels were significantly associated with the likelihood of all-cause and cardiovascular mortality in individuals with CHD, but also that this association exhibited a linear negative correlation pattern. These results help to define the effects of serum albumin on CVD, and remind us of the importance to monitor serum albumin levels in patients with CHD to improve the adverse outcomes of patients with CVD.

## Availability of Data and Materials

All raw data used in this study can be accessed on the NHANES website (https://wwwn.cdc.gov/nchs/nhanes/default.aspx).
